# Prevention of health care associated venous thromboembolism through implementing VTE prevention clinical practice guidelines in hospitalized medical patients: a systematic review and meta-analysis

**DOI:** 10.1186/s13012-020-01008-9

**Published:** 2020-06-24

**Authors:** Juliana Abboud, Abir Abdel Rahman, Lara Kahale, Martin Dempster, Pauline Adair

**Affiliations:** 1 Centre for Improving Health Related Quality of Life, School of Psychology, Queens University Belfast, David Keir Building, 18-30 Malone Road, Belfast, BT9 5BN UK; 2 Department of Medical Laboratory Sciences, Faculty of Health Sciences, University of Balamand, Ashrafieh, Youssef Sursok Street, PO Box 166378, Beirut, Lebanon; 3 AUB GRADE Center, Clinical Research Institute, American University of Beirut, Academic and Clinical Center (ACC), 3rd floor, Riad El Solh, PO Box: 11-0236, Beirut, 1107 2020 Lebanon

**Keywords:** Thromboprophylaxis, Venous thromboembolism, Guidelines implementation, Risk assessment, Prophylaxis, Medical patients

## Abstract

**Background:**

Venous thromboembolism (VTE) is a leading cause of morbidity and mortality in hospitalized patients. Numerous VTE prevention clinical practice guidelines are available but not consistently implemented. This systematic review explored effectiveness of implementing VTE prevention clinical practice guidelines on VTE risk assessment and appropriateness of prophylaxis in hospitalized adult medical patients and identified the interventions followed to improve the adherence to these guidelines.

**Methods:**

Six electronic databases were searched for randomized controlled trials, clinical controlled trials, or pre/post evaluation studies up to January 2019. Studies identified were screened for eligibility by two reviewers independently. Data were extracted by two reviewers using a standardized form. Risk of bias was assessed using MINORS and the certainty of evidence for each outcome using the GRADE approach.

**Results:**

Of the 3537 records identified, 36 were eligible; eight studies were included for qualitative synthesis and four for meta-analysis. The meta-analysis of the studies assessing the impact of implementing VTE clinical practice guidelines favored appropriate prophylaxis (RR 1.67, 95% CI 1.41 to 1.97, 552 patients). Potential risk of bias was assessed to be low for 28% of the studies. However, using GRADE, the certainty of the evidence of all outcomes was rated very low quality.

**Conclusions:**

The lack of randomized controlled trials in this area reduces the quality of the evidence available. The evidence from before-after studies suggests that the implementation of VTE clinical practice guidelines may increase the practice of VTE risk assessment and appropriate prophylaxis in hospitalized medical patients.

**Trial registration:**

PROSPERO CRD42018085506

Contributions to the literature

- Current evidence-based guidelines for VTE prevention recommend the use of risk assessment to identify the appropriate prophylaxis regimens for different levels of risk. Research has shown that many hospitalized patients do not receive venous thromboembolism (VTE) prophylaxis despite being assessed as being at risk of VTE.

- The findings of this review support the ongoing need to implement guidelines to improve patient health outcomes.

- VTE prophylaxis in medical hospitalized patients, including ascertaining how, when, and what VTE clinical practices guidelines are used in studies, will improve the compliance of appropriate prophylaxis in hospitals.

## Background

Clinical practice guidelines are evidence-based recommendations that support clinicians in making decisions about patient care within specific conditions. The Institute of Medicine (IOM) defines clinical practice guidelines as “statements that include recommendations, intended to optimize patient care, that are informed by a systematic review of evidence and an assessment of the benefits and harms of alternative care options” [1].

Such guidelines help to standardize medical and surgical care, reduce variations in clinical practice, improve the quality and consistency of care, increase the efficiency in health care services, weigh the risks and benefits to guide treatment decisions, and promote patient engagement in care management [2–4].

Venous thromboembolism is a medical condition that is addressed in many clinical practice guidelines. Venous thromboembolism (VTE) is a leading cause of morbidity and mortality in hospitalized patients. VTE is a blood clot that most frequently starts in the deep veins of the legs or pelvis, a deep vein thrombosis (DVT), when the blood clot or part of it breaks free from a vein wall and travels to the lungs where it can block some or all of the blood supply, called a pulmonary embolism (PE), which in some cases can be fatal [5].

Numerous evidence-based guidelines outline the appropriate prophylaxis to prevent venous thromboembolism (VTE) in hospitalized patients [6–10]. Yet, despite the existence of these guidelines, VTE events and VTE-related deaths still occur in hospitals. It has been reported that around 500,000 VTE events occur in the USA annually [11] and an estimate of more than 600,000 DVT events and 400,000 PE events across the European Union [12].

Moreover, it was revealed that 52% of the annually reported VTE in USA were related to current or recent hospitalization where 25% of hospitalization-related events occurred during an inpatient stay and 75% of these events happened within 92 days of hospital discharge, with a median of 19.5 days [13].

Furthermore, many hospitalized patients diagnosed with VTE die each year. It is estimated that an average of 28,726 hospitalized patients diagnosed with VTE die each year in the USA, and three quarters of the 500,000 VTE-related deaths were from hospital-acquired VTE in Europe. Consequently, different strategies and initiatives have been implemented in different countries to improve VTE prevention practices. For example, in the USA, a call to action was issued by the surgeon general to reduce VTE in 2008 [14]; the Agency for Healthcare Research and Quality developed a guide to support hospitals in implementing VTE prevention initiatives [15] and considered VTE prevention a top patient safety priority [16]; the National Institute for Clinical Excellence introduced a quality standard that covers reducing the risk of venous thromboembolism for use in all UK hospitals [10]; the Australian Commission on Safety and Quality in Health Care implemented a VTE prevention program [17]. In spite of these, there was no decrease over time in the incidence of VTE nor the death cases from PE in hospitalized patients [18, 19].

It is clear therefore that there is a gap between the evidence and clinical practice.

Although available, VTE prevention clinical practice guidelines and the recommendations for VTE prophylaxis in medical patients, as described in the guidelines (see Additional file 1 for VTE guidelines), are not properly followed. A cross-sectional study conducted across 32 countries showed that only 39.5% of medical patients at risk of VTE had received appropriate prophylaxis compared to 58.5% of surgical patients. The medical patients’ appropriate prophylaxis compliance rate varied from 3 to 70% between countries [20]. In Europe and the Middle East, studies revealed that prophylaxis was administered less to medical patients than surgical patients although medical patients had higher risk of developing VTE based on the VTE risk assessment [21–24].

The aim of the present systematic review was to assess the effectiveness of implementing VTE clinical practice guidelines on VTE risk assessment and appropriateness of prophylaxis in hospitalized adult medical patients and to identify interventions that modify the health care provider’s behavior and improve their adherence to implementing VTE clinical practice guidelines. Moreover, this systematic review explored several areas of VTE prevention clinical practice guidelines in hospitalized medical patients including what guidelines exist, their quality, and VTE risk assessment tools used.

## Methods

The systematic review is reported in accordance with Preferred Reporting Items for Systematic Reviews and Meta-Analysis (PRISMA) 2009 checklist [25, 26] (see Additional file 2 for the checklist).

A protocol was developed and registered with the International Prospective Register of Systematic Reviews (PROSPERO) on 7 February 2018 (Registration number: CRD42018085506) [27].

### Eligibility criteria

Randomized controlled trials (RCTs), controlled trials, and pre/post design studies were included in this study, and English language articles only were eligible.

Studies were eligible for inclusion if they evaluated the effectiveness of implementing the VTE clinical practice guidelines for hospitalized adult medical inpatients with medical conditions (e.g., respiratory, diabetes, infectious diseases). On the other hand, studies were excluded if they focused on the treatment of VTE in non-medical, surgical, cancer, or obstetric patients or patients aged less than 18 years. Studies involving patients from mixed specialties (e.g., medical as well as surgical patients) were included if it was possible to extract the data solely pertaining to medical patients.

Outcome measures for this review were risk assessment, which is measured as the proportion of patients who were assessed for risk of VTE as per the guidelines; appropriate prophylaxis, identified as the proportion of patients who received appropriate prophylaxis as per the guidelines; and inappropriate prophylaxis, which is presented as the proportion of patients who (1) had no indication but prophylaxis given and (2) had an indication but prophylaxis was not given.

### Search strategy

The search included papers in the English language only.

The following databases were searched from their inception until January 2019: CINAHL Cumulative Index of Nursing and Allied Health Literature (accessed via EBSCO); Cochrane Central Register of Controlled Trials; EMBASE (accessed via OVID); MEDLINE: (accessed via OVID); Pubmed (accessed via OVID); and SCOPUS (accessed via EBSCO).

Google scholar was searched to obtain additional publications. In addition, reference lists of all articles included at the final stage in the review were checked to locate further relevant titles. Searches in all the above databases were executed using the appropriate keywords. All related MeSH terms, indexed words, or indexed mapped terms were explored in the selected databases (see Additional file 3 for search terms and Search strategy performed in each database). All retrieved citations were downloaded and managed using the End Note software. In addition, reviewers attempted to contact authors for identified incomplete reported data.

### Data extraction and analysis

#### Selection of studies

All retrieved titles and abstracts were examined for relevance by two reviewers (JL, AA). The two reviewers independently screened the titles and abstracts of identified articles. Full text of articles judged as potentially eligible by at least one of the reviewers were retrieved. They also independently read the full-text of all included studies to check eligibility using a standardized data extraction screening form.

A consensus meeting to discuss studies that had not reached agreement was planned to be undertaken by the two main reviewers where required, and if unresolved, a third review author (PA) would be consulted.

The agreement between both reviewers was assessed for study inclusion using the Kappa statistic [28]. Kappa values were interpreted as follows: 0 to 0.20 represented slight agreement, 0.21 to 0.40 fair agreements, 0.41 to 0.60 moderate agreements, 0.61 to 0.80 substantial agreements, and greater than 0.80 almost perfect agreements.

#### Data extraction

Two reviewers (JL and AA) independently performed data extraction from each included study. The data collected included (1) study ID (surname of first author and year first full report of study was published); (2) study design (RCTs, clinical controlled trial, or pre/post evaluation); (3) setting (hospital services, adult medical patients); (4) measurement/intervention (guidelines followed, risk assessment tool used, and other interventions implemented to improve the adherence to VTE guidelines); (5) outcome measures (the proportion of patients who were assessed for risk of VTE, the proportion of patients who received appropriate prophylaxis, the proportion of patients who had no indication but prophylaxis given, and the proportion of patients had an indication, but prophylaxis was not given); and (6) overall conclusion.

### Risk of bias

The risk of bias was assessed at the study level, and checklists chosen were relevant to study designs. MINORS was chosen as a methodological index for non-randomized studies [29, 30] and the Cochrane risk-of-bias tool for identified RCT studies (see Additional file 6 for MINORS). Two reviewers (JA and AA) independently assessed the methodological quality of each of the included studies. They resolved disagreements by discussion. Persistent discrepancies were resolved by discussion with the third reviewer (PA).

Risk of bias for RCT studies was assessed according to the following criteria: random sequence generation (selection bias), allocation concealment (selection bias), blinding of participants and personnel (performance bias), blinding of outcome assessment (detection bias), incomplete outcome data (attrition bias) and selective reporting, and whether the study was free of selective outcome reporting (reporting bias).

### Data synthesis

For dichotomous outcomes, the risk ratio (RR) with 95% confidence intervals (CIs) was calculated. The intention to treat principle was applied; then, the results of the different studies were pooled using a random-effects model. The certainty of evidence at the outcome level was assessed using the GRADE approach [31]. None of the outcome measures were continuous variables. A narrative synthesis of the characteristics of the included studies and a forest plot of study findings are presented. Data across studies were pooled where possible, but high levels of heterogeneity were anticipated.

Heterogeneity between studies was assessed by visual inspection of forest plots and by estimation of the percentage heterogeneity between studies that was not due to chance (*I*^2^ test) [32]. If there was evidence of substantial heterogeneity, an attempt to investigate the possible reasons for it was done. If test subgroup effect was significant, the pooled meta-analysis was not reported, instead the reporting should be on the results of the individual studies. In this case, definitive conclusions cannot be drawn until more studies become available [33].

## Results

### Descriptions of studies

#### Results of the search

**Fig. 1:**
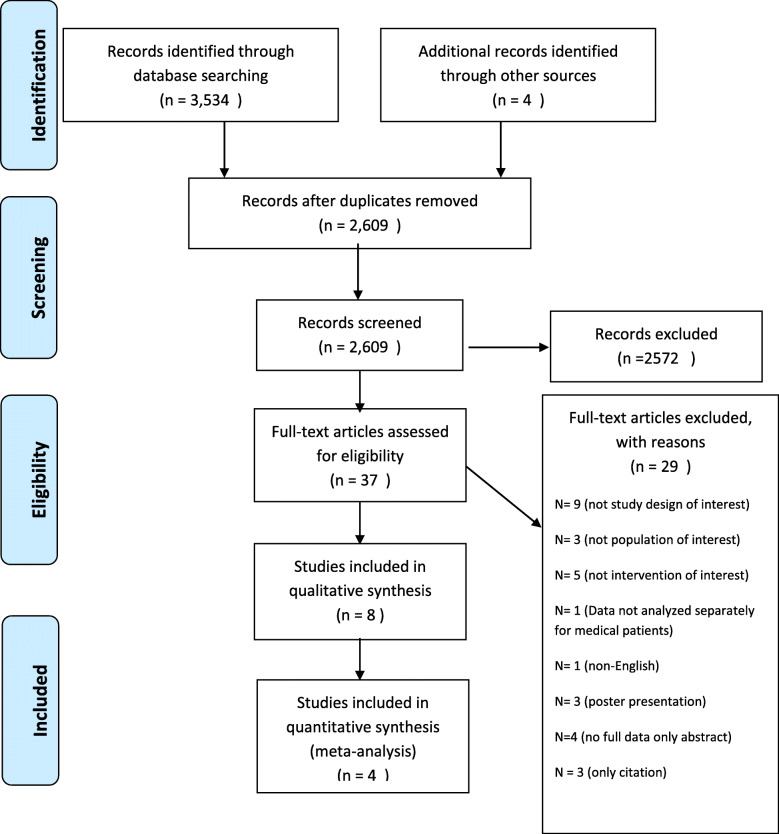
Flow diagram of the literature search process

Thirty-seven studies were included from the 3538 records originally identified (see Fig. 1). Eight studies [34–41] were included in the qualitative synthesis, of which only 4 studies [34, 35, 37, 41] were included in the meta-analysis. Agreement between authors on study eligibility during the full-text screening was found to be high (kappa = 0.85).

#### Study characteristics

The included studies involved 33,876 medical patients; the median was 303 medical patients, and the length of study period ranged from 16 weeks [36] to 4 years [39]. All selected studies were published in English (see Additional file 4 for characteristics of included studies). Of the eight selected studies, three were undertaken in Europe (2 in UK, one in Italy) [38, 40, 41] and one each in the USA [37], Canada [36], Australia [34], Brazil [39], and Iran [35]. Patients were recruited from acute hospitals ranging from 250 to 1500-bed hospital [34, 41], and two studies included a group of hospitals [36, 40]. Pre- and post-study design was common for all included studies except one study that used a cluster randomized controlled trial [36]. Four studies assessed only medical patients [35, 36, 38, 40], and the other four targeted both medical and surgical patients; however, data was presented for medical patients separately [34, 37, 39, 41].

#### VTE prevention clinical practice guidelines implemented in the studies

The 8th American College of Chest Physicians (ACCP) Evidence-Based Clinical Practice Guidelines [42] was used by 2 studies [36, 39]. One study [34] audited compliance with the Best Practice Guidelines for Australia and New Zealand 4th edition [43]. Another study [40] followed the recommendations of both the thromboembolic risk factor (THRIFT) and 7th American College of Chest Physicians (ACCP) consensus groups [44, 45]. Moreover, a study [37] implemented the VTE prophylaxis regimens adapted from Caprini’s VTE risk assessment tool [46]. In three studies [35, 38, 41], local guidelines were developed; one of them [38] adopted the risk factors for VTE from the THRIFT consensus group [45] and the contraindications and cautions to thromboprophylaxis from the Scottish Intercollegiate Guidelines Network (SIGN) 62 [6]. In another study [41], the locally adapted guidelines were based on the American College of Chest Physicians (ACCP) 2001 recommendations [47], and it was reviewed by three external experts before dissemination. The third study [35] followed a consensus method prepared internally by clinical pharmacists; however, no review or validation was reported by the study.

#### VTE risk assessment tools used in the studies

VTE risk assessment implemented in different studies [34, 36, 39, 41] was based on the risk factors as per the adopted guidelines. VTE risk stratifications according to the (TRIFT) consensus group risk factors were followed in two studies [38, 40]. In one study [35], the risk points for thrombosis risk assessment were determined by an internal institutional agreement. Only one study [37] used a thrombosis risk assessment tool following a risk scoring system, the Caprini. This risk assessment model was validated in many publications [48–54].

#### Interventions followed in the studies to modify the health care providers’ behavior

To facilitate the implementation of the VTE guidelines and improve the rate of appropriate prophylaxis, a variety of interventions were used in the studies (see Additional file 4 for characteristics of included studies). Three studies used single intervention strategies by either placing the VTE risk assessment in the patient’s chart [37] or presenting the recommended guidelines and data to clinicians [35, 40]. The percentage of patients receiving appropriate prophylaxis increased in these three studies after intervention, and it ranged between 31 [35, 40] and 76% in Shedd et al. who reported the highest improvement rate between all studies (33%).

On the other hand, five studies [34, 36, 38, 39, 41] implemented multi component interventions strategies including audit and feedback, staff meetings and presenting compliance results, education and training, documentation aids, policy and procedures, and alerts and reminders. At least, a combination of three interventions was used to increase the uptake of VTE prophylaxis guidelines. Most of these studies showed improvement results in measuring the desired outcomes. The medical patients’ appropriate prophylaxis compliance rate varied from 31 to 72% between studies after intervention implementation. The only randomized controlled trial study in this review [36] reported no significant difference in appropriate VTE prophylaxis rates between intervention and control hospitals.

Audit and feedback was the most commonly reported intervention that supported the implementation of VTE guidelines. All but one of the studies in the review had an audit and feedback element including meetings to illustrate the results, presentations, and audit and feedback sessions [34–36, 38–41]. Four studies [34, 36, 38, 39] reported on conducting education and training to inform the implementation of the VTE guidelines. Five interventions [34, 36–38, 41] focused on documentation procedures such as providing decision support aids, pocket version of the guideline, paper-based VTE assessment algorithm, and printed physicians’ orders. Hospital and VTE prevention policy were highlighted only in two papers [34, 39] and alerts and reminders too [34, 41]. One study [34] explored the barriers to inform the intervention strategies; it identified strategies through literature review and brain stormed possible barriers to VTE guideline uptake, and accordingly four strategies for change were selected. While another study [36] focused on formal feedback sessions, as well as paper and electronic questionnaires distributed to healthcare providers at the intervention hospitals at the end of the study to identify data to improve the implementation of the VTE risk assessment form.

#### Excluded studies

Twenty-nine studies were excluded from the review with reasons for exclusion summarized in (see Additional file 5 for Reasons for excluded studies).

#### Risk of bias in included studies

The quality of the eight studies included in the review was evaluated. Among the eight studies included in this review, Pai was the only study identified with a randomized controlled trial design. The quality of evidence of this study was assessed separately using the Cochrane tool for risk of bias. This study scored “low risk” in the domains related to selection bias (random sequence generation). It scored high in the domain related to performance bias which includes blinding of participants and personnel. In addition, the risk of bias was unclear in relation to attrition bias, reporting bias, and other risk of bias.

Upon applying the MINORS methodological index for grading of the seven included non-comparative articles, the resulting scores ranged from 10/16 to 16/16 for the eight domains included in the adopted tool for non-comparative studies. The mean and median scores were 12.2 and 12, respectively. One study had a MINORS score of 16/16 and was considered to have a low risk of bias while the other studies scores ranged from 10/16 to 13/16 and were reported with high risk of bias. In brief, all studies assessed with MINORS had clearly stated the aim, inclusion of consecutive patients, and prospective collection of data. Quality assessment was conducted independently by two reviewers (JA and AA) and achieved a uniform bias assessment.

Detailed risk of bias assessment using MINORS for included studies in this review are presented in Additional file 6 for Summary of risk of bias-MINORS.

### Effects of VTE guidelines implementation

#### Risk assessment for VTE

Two studies [34, 39] reported on VTE risk assessment among medical patients pre and post VTE clinical practice guidelines implementation. One of these studies [39] contributed 100% showing that the implementation of VTE guidelines may increase risk assessment practices at 1 year; however, the chance of a VTE guideline improving risk assessment practice is very small. The certainty of evidence for risk assessment was very low due to observational study design and serious risk of bias and given the difference in contribution between the two studies; pooling the data from both was not undertaken.

#### Appropriate prophylaxis

**Fig. 2:**
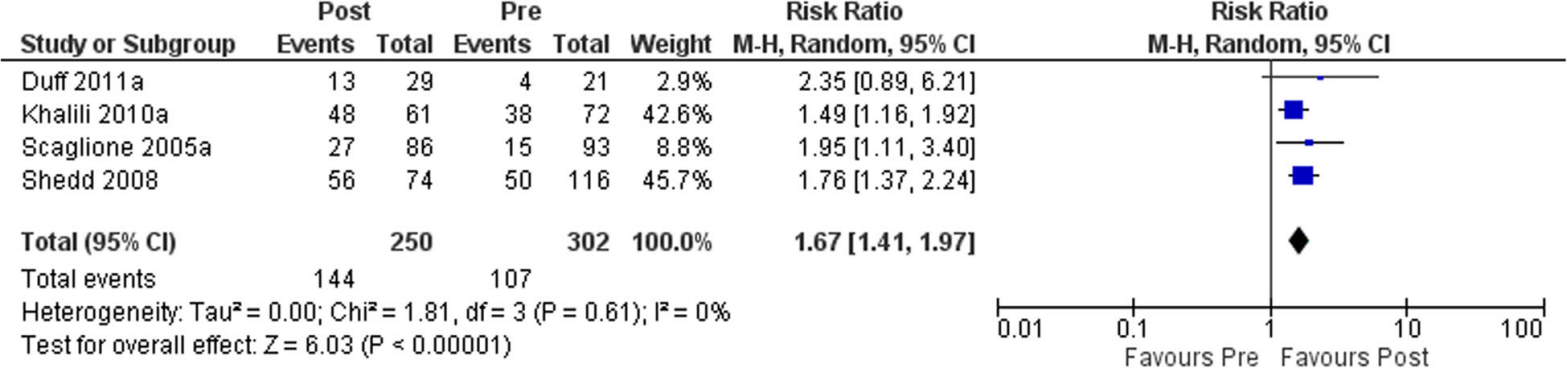
Forest plot showing the effect of the implementation of VTE guidelines on appropriate prophylaxis

The meta-analysis of four studies [34, 35, 37, 41], including 552 patients, and comparing appropriate prophylaxis pre and post implementation of VTE clinical practice guidelines in hospitalized medical patients showed a statistically significant effect on appropriate prophylaxis (RR 1.67, 95% CI 1.41 to 1.97) (see Fig. 2). Thus, VTE clinical practice guideline implementation may increase the appropriate prophylaxis. The *I*^2^ value indicated that the percentage of the variability in effect estimates that was due to heterogeneity rather than sampling error (chance) was absent (*I*^2^ = 0%). In a subgroup analysis of participants with high risk versus non-high risk, the test for subgroup effect was not statistically significant (*p* value 0.96), indicating no subgroup effect of the level of baseline risk. The certainty of evidence for appropriate prophylaxis was very low due to observational study design and serious risk of bias.

#### No indication but prophylaxis given

One study [35], including 207 patients, comparing appropriate prophylaxis pre and post implementation of VTE clinical practice guidelines in hospitalized medical patients may reduce inappropriate prophylaxis, i.e., prophylaxis given when not indicated (RR 0.28; 95% CI 0.11 to 0.71). The certainty of evidence for inappropriate prophylaxis, i.e., prophylaxis given when not indicated, was very low due to observational study design and serious risk of bias.

One study [40], including 1062 medical patients, reported a decrease in the percentage of medical patients at moderate or high risk receiving incorrect VTE prophylaxis. The percentage decreased from 95.6 to 69.3% based on THRIFT consensus group and from 78.3 to 69.3% according to the ACCP guidelines.

#### Indication but prophylaxis not given

One study [35] including 47 patients, and comparing appropriate prophylaxis pre and post implementation of VTE clinical practice guidelines in hospitalized medical patients, found a significant decrease in inappropriate prophylaxis—prophylaxis not given when indicated (RR 0.45, 95% CI 0.26 to 0.77). The certainty of evidence for inappropriate prophylaxis, i.e., prophylaxis not given when indicated, was very low due to observational study design and serious risk of bias.

The three studies that followed the American College of Chest Physicians (ACCP) clinical practice guidelines had the lowest improvement rate; it ranged between 2.1 and 10%. Based on the Australian and New Zealand guidelines, one study [34] demonstrated a 25.8% improvement rate in appropriate prophylaxis, and guidelines prepared internally [35] had a less improvement rate of 10.8% compared to other studies in this review. Moreover, it was noted when using a more focused approach on individual recommendations within the guideline, such as conducting VTE risk assessment, improved more the compliance with appropriate prophylaxis. This was identified in studies who focused their interventions on implementing the risk assessment tool and had the highest improvement rate in appropriate prophylaxis, where Shedd et al. study improved by 33% [37], Duff et al. by 25.8% [34], and Vaughan et al. study by 32% [38] for moderate- and high-risk patients.

The Pai study (the only RCT) [32] found no significant difference between the rates of appropriate thromboprophylaxis between groups (OR = 0.80 in intervention versus control groups, 95% CI).

## Discussion

Venous thromboembolism (VTE) is a leading cause of morbidity and mortality in hospitalized patients. Although VTE prevention guidelines are available, they are not consistently implemented. An improvement in appropriate prophylaxis was associated with the implementation of VTE guidelines

On the other hand, conducting VTE risk assessment is an important part of the VTE prevention clinical practice guidelines. Several studies have reported that a reduction in hospital acquired VTE events is significantly associated with the introduction of the risk assessment [55, 56]. A call to action was initiated during World Thrombosis Day in 2014 to make VTE risk assessment a priority since the use of VTE risk assessment reduces the occurrence of VTE [57]. Moreover, the National Health Service in England supported routine VTE risk assessment in hospitalized patients to reduce death and morbidity from VTE. This was done through making the risk assessment tool available to all National Health Service Providers in England in 2010 along with the National Institute for Health and Care Excellence (NICE) recommendations that “All patients, on admission, receive an assessment of VTE and bleeding risk using the clinical risk assessment criteria described in the national tool.” According to the official statistics for VTE risk assessment in England, the percentage of VTE risk assessments on adult inpatients admitted to NHS-funded acute care increased from 53% in Q2 (2010/11) to 96% in Q4 (2018/19). An improvement trend was observed since mandatory risk assessment was introduced in England [9, 58, 59].

The findings from this review point towards making risk assessment a priority as well as focusing on single behaviors to improve the outcomes. The focus should be on risk assessment as the most important behavior to target as it precedes administration of prophylaxis. Evidence suggests it is more challenging to change multiple behaviors than single behavior thus the need to focus future interventions on the implementation of the risk assessment.

This is also comparable to a previous systematic review on the use and effectiveness of guidelines in practice; it showed that changing behavior interventions should focus on single guideline recommendation rather than targeting all guideline statements. The interventions should be tailored to single targeted behavior that needs to be improved. This approach could improve more the use and effectiveness of guidelines in practice [60].

This systematic review did not identify any study that used a behavior change framework to inform the intervention to increase the VTE guidelines uptake. Hence, there is a need to explore this behavior using the appropriate behavior change model for researchers to develop focused and tailored interventions. In addition, there is an ultimate need to develop interventions grouping medical patients different from interventions grouping surgical patients.

On the other hand, the majority of studies included in the current review used multifaceted strategies, such as audit and feedback, presenting compliance results, education, documentation aids, alerts, and reminders to improve VTE guidelines implementation which is similar to a previous systematic review of 55 studies which implemented strategies to improve thromboprophylaxis rates in hospitalized medical and surgical patients. Different interventions can be more effective in improving the rate of prescribing appropriate prophylaxis than only one intervention [61]. Moreover, a literature review about the methods to improve prophylaxis reported that a passive guideline dissemination, by only distributing and communicating the guideline, is unlikely to improve VTE prophylaxis and should not be used alone it should be reinforced by other interventions such as education and monitoring [62]. However, the multi component nature of the majority of implementation strategies did not allow for a clear identification of the effectiveness of individual strategies. Moreover, it was difficult to assess if multiple components were more efficient than a single intervention. Identifying the active ingredients of behavior change interventions is a recognized problem for complex interventions, yet the importance of itemizing the mechanisms of change is well supported in the literature [63, 64]. While much work has been undertaken over the past 10 years to support the identification of behavior change interventions, not all areas of study have embraced this, and there is much more work to be done especially where implementing guidelines are concerned. The use of checklists has provided some support, and it is recommended that interventions to improve implementation of guidelines is better reported so that the aspects of the multi-modal intervention that work are understood [65, 66].

Moreover, future studies could focus on assessing the effectiveness of guidelines over time; a longer term follow-up period is needed since the intervention period might affect the improvement rate. This was observed in one study [39] initiated in 2010 and after 1 year of implementation, the appropriate prophylaxis increased from 62 to 72%. However, after 8 years, it reached 85% and the percentage of risk assessment 98.03%.

## Strengths and limitations

This systematic review has a number of strengths. Research procedures are reported in detail in the protocol, which will enhance transparency and facilitate the update of this systematic review by future reviewers. The literature review was exhaustively explored. It includes rigorous methodology and explicit, broad eligible criteria aiming at high sensitivity. A systematic approach was used to study selection, data abstraction, and data synthesis with two independent reviewers. In addition, this review included studies with multi-component interventions conducted in hospitals in different countries. Furthermore, the various characteristics of the studies, wide range of VTE prophylaxis guidelines, and risk assessment tools were explored. Moreover, the overall completeness of the data allowed the inclusion of 4 out of 8 eligible studies in the meta–analysis.

On the other hand, findings from the systematic review need to be considered in light of several limitations. The uncontrolled before and after design is a limitation of the review as only one study adopted a cluster randomized controlled design. Moreover, there is no standardized risk of bias assessment method for the variation of study designs included in this review. Thus, we used the MINORS risk of bias criteria which are relevant to non-randomized studies. Studies with full MINORS score were considered to have low risk bias, and high risk of bias was considered in studies with incomplete score. In addition, many papers were not included in this systematic review, as in some studies data related to medical patients was not reported separately from surgical patients and therefore not included. Furthermore, four out of the eight included studies for review were only targeting medical patients. Some studies did not address which risk assessment tool was followed; therefore, the variability across studies may be greater. The studies included in this review provided a wide range of multifaceted approaches which could be optimal for one particular setting and not appropriate in another setting. This review may have been subject to clinical heterogeneity. This could be attributed to variability in settings and health professionals involved from different countries with diversified cultures. The search strategy was limited by studies in the English language which may potentially have resulted in the failure to identify all relevant studies.

## Conclusion

Findings from this systematic review indicate that implementation of clinical practice guidelines increases the percentage of hospitalized medical patients who are assessed for VTE risk and prescribed appropriate prophylaxis. However, the lack of randomized controlled trials in this area reduces the quality of the evidence available. In view of devising interventions for clinicians, the following guidelines are suggested for reviewers and researchers: (1) develop well designed, prospective cohort studies and RCTs to increase the pool of studies that might report on VTE guidelines in medical patients; (2) focus on the explicit implementation of the VTE risk assessment and the length of the intervention to reinforce the implementation of the VTE guidelines; (3) explore which theories and mediators of change can increase VTE guidelines implementation effectiveness; and (4) utilize standardized outcome measures to assist researchers in improving their protocols and minimizing risk of bias.

## Supplementary information

**Additional file 1.** VTE guidelines recommendations for medical patients.

**Additional file 2.** PRISMA 2009 Checklist.

**Additional file 3.** Search terms. Search strategy performed in each database.

**Additional file 4.** Characteristics of included studies.

**Additional file 5.** Reasons for excluded studies.

**Additional file 6.** MINORS Summary of risk of bias.

## Data Availability

All data generated or analyzed during this study are included in this published article and additional files including articles included in the analysis which are cited in the reference list.

## Abbreviations

VTE: Venous thromboembolism
PE: Pulmonary embolism
DVT: Deep vein thrombosis
RCT: Randomized controlled trial
