# Supplementary material for: Prevention of health care associated venous thromboembolism through implementing VTE prevention clinical practice guidelines in hospitalized medical patients: a systematic review and meta-analysis
Source: Implement Sci. 2020 Jun 24;15:49. doi: 10.1186/s13012-020-01008-9 (PMC7315522; doi:10.1186/s13012-020-01008-9)
Supplement: Supplementary file 1 — Additional file 1. VTE guidelines recommendations for medical patients. [file 13012_2020_1008_MOESM1_ESM.docx]

| **Major Guidelines Addressing VTE Prophylaxis in medical patients** | | **Date** | **Applicable to** | **Recommendations / Grades of recommendation** |
| --- | --- | --- | --- | --- |
| National Institute for Health and Clinical Excellence,  NICE | Venous thromboembolism in over 16s: reducing the risk of hospital-acquired deep vein thrombosis or pulmonary embolism | Published March 2018  Updated:  August 2019 | All patients | **Medical patients recommendations**   - Assess all medical patients to identify the risk of VTE and bleeding: - Balance the person's individual risk of VTE against their risk of bleeding when deciding whether to offer pharmacological thromboprophylaxis to medical patients. - If using pharmacological VTE prophylaxis for medical patients, start it as soon as possible and within 14 hours of admission, unless otherwise stated in the population-specific recommendations   **Reassessment of risk of VTE and bleeding**   - Reassess all medical, surgical and trauma patients for risk of VTE and bleeding at the point of consultant review or if their clinical condition changes.   **(GRADE) approach for appraising evidence** |
| Scottish Intercollegiate Guidelines Network  (SIGN) | Prevention and management of venous  thromboembolism | Published December 2010  Updated 2014 | All patients | - - All patients admitted to hospital or presenting acutely to hospital should be individually assessed for risk of venous thromboembolism and bleeding. The risks and benefits of prophylaxis should be discussed with the patient.   - The use of a risk assessment method checklist is recommended for this purpose.   - The assessment should be repeated regularly and at least every 48 hours.   **Grade D**  Evidence level 3 or 4; or Extrapolated evidence from studies rated as 2+ GOOD |
|  |  |  |  | **Medical patients recommendations**   - - When the assessment of risk favours use of thromboprophylaxis, unfractionated heparin, low molecular weight heparin or fondaparinux should be administered.   **Grade A**  At least one meta-analysis, systematic review, or RCT rated as 1++,  and directly applicable to the target population; or A body of evidence consisting principally of studies rated as 1+, directly applicable to the target population, and demonstrating overall consistency of results |
| American College of Chest Physicians (ACCP) | Prevention of VTE in nonsurgical patients: antithrombotic therapy and prevention of thrombosis, 9th ed: American College of Chest Physicians evidence-based clinical practice guidelines. | Published 2012 | Nonsurgical Patients | - For acutely ill hospitalized medical patients at increased risk of thrombosis, we recommend anticoagulant thromboprophylaxis with low-molecular-weight heparin [LMWH], low-dose unfractionated heparin (LDUH) bid, LDUH tid, or fondaparinux **(Grade 1B)** - For acutely ill hospitalized medical patients at low risk of thrombosis, we recommend against the use of pharmacologic prophylaxis or mechanical prophylaxis **(Grade 1B)** - For acutely ill hospitalized medical patients who are bleeding or at high risk for bleeding, we recommend against anticoagulant thromboprophylaxis **(Grade 1B).** - For acutely ill hospitalized medical patients at increased risk of thrombosis who are bleeding or at high risk for major bleeding, we suggest the optimal use of mechanical thromboprophylaxis with graduated compression stockings (GCS) **(Grade 2C)** or intermittent pneumatic compression (IPC) **(Grade 2C),** rather than no mechanical thromboprophylaxis. When bleeding risk decreases, and if VTE risk persists, we suggest that pharmacologic thromboprophylaxis be substituted for mechanical thromboprophylaxis **(Grade 2B).** - In acutely ill hospitalized medical patients who receive an initial course of thromboprophylaxis, we suggest against extending the duration of thromboprophylaxis beyond the period of patient immobilization or acute hospital stay **(Grade 2B).**   **(GRADE) approach for appraising evidence** |
| American College of Physicians  ACP | Venous Thromboembolism Prophylaxis in Hospitalized Patients: A Clinical Practice Guideline From the American College of Physicians (ACP). | Published  2011 | Nonsurgical patients. Medical and stroke. | - **Recommendation 1:** ACP recommends assessment of the risk for thromboembolism and bleeding in medical (including stroke) patients prior to initiation of prophylaxis of venous thromboembolism **(Grade: strong recommendation, moderate-quality evidence).** - **Recommendation 2:** ACP recommends pharmacologic prophylaxis with heparin or a related drug for venous thromboembolism in medical (including stroke) patients unless the assessed risk for bleeding outweighs the likely benefits **(Grade: strong recommendation, moderate-quality evidence).** - **Recommendation 3:** ACP recommends against the use of mechanical prophylaxis with graduated compression stockings for prevention of venous thromboembolism **(Grade: strong recommendation, moderate-quality evidence)**   **(GRADE) approach for appraising evidence** |
| American College of Chest Physicians (ACCP) | Antithrombotic Therapy and Prevention of Thrombosis, 8th ed: American College of Chest Physicians (ACCP) Evidence-Based Clinical Practice Guidelines. | Published 2008 | Medical and surgical patients | - For every general hospital, we recommend that a formal, active strategy that addresses the prevention of VTE be developed **(Grade 1A).** - We recommend that the local thromboprophylaxis strategy be in the form of a written, institution-wide thromboprophylaxis policy **(Grade 1C).** - We recommend the use of strategies shown to increase thromboprophylaxis adherence, including the use of computer decision support systems **(Grade 1A),** preprinted orders **(Grade 1B),** and periodic audit and feedback **(Grade 1C).** Passive methods such as distribution of educational materials or educational meetings are not recommended as sole strategies to increase adherence to thromboprophylaxis **(Grade 1B).**   **Medical patients recommendations**   - In patients admitted to hospital with an acute medical illness, we recommend thromboprophylaxis with LMWH, LDUH, or fondaparinux **(each Grade 1A).** - For medical patients with risk factors for VTE, and for whom there is a contraindication to anticoagulant thromboprophylaxis, we recommend the optimal use of mechanical thromboprophylaxis with GCS or IPC **(Grade 1A).**   **(GRADE) approach for appraising evidence** |
