# Supplementary material for: Prevention of health care associated venous thromboembolism through implementing VTE prevention clinical practice guidelines in hospitalized medical patients: a systematic review and meta-analysis
Source: Implement Sci. 2020 Jun 24;15:49. doi: 10.1186/s13012-020-01008-9 (PMC7315522; doi:10.1186/s13012-020-01008-9)
Supplement: Supplementary file 3 — Additional file 3. Search terms. Search strategy performed in each database. [file 13012_2020_1008_MOESM3_ESM.docx]

OVID Databases (Embase, Medline)

| 1. medical.mp. or medical care/ |  |
| --- | --- |
| 2. hospitalized.mp. |  |
| 3. high risk patient/ or patient/ or high risk.mp. |  |
| 4. at risk.mp. or risk assessment/ |  |
| 5. 1 or 2 or 3 or 4 |  |
| 6. patients.mp. or patient/ |  |
| 7. inpatients.mp. or hospital patient/ |  |
| 8. 6 or 7 |  |
| 9. 5 and 8 |  |
| 10. clinical practice guidelines.mp. or practice guideline/ |  |
| 11. evidence based practice.mp. or evidence based medicine/ or evidence based practice/ |  |
| 12. bundles.mp. or care bundle/ |  |
| 13. clinical pathway/ or pathways.mp. |  |
| 14. protocols.mp. or clinical protocol/ |  |
| 15. guidelines.mp. |  |
| 16. 10 or 11 or 12 or 13 or 14 or 15 |  |
| 17. venous thromboembolism.mp. or venous thromboembolism/ |  |
| 18. lung embolism/ or thromboembolism/ or venous thromboembolism/ or prophylaxis/ or VTE.mp. or deep vein thrombosis/ |  |
| 19. VTE prophylaxis.mp. |  |
| 20. 17 or 18 or 19 |  |
| 21. 16 and 20 |  |
| 22. prophylaxis.mp. or prophylaxis/ or antibiotic prophylaxis/ |  |
| 23. prophylactic.mp. |  |
| 24. prevent.mp. |  |
| 25. reduction.mp. |  |
| 26. decrease.mp. |  |
| 27. diminish.mp. |  |
| 28. 22 or 23 or 24 or 25 or 26 or 27 |  |
| 29. adherence.mp. |  |
| 30. compliance.mp. |  |
| 31. 29 or 30 |  |
| 32. 28 and 31 |  |
| 33. 9 and 21 and 32 |  |

| medical.mp. |  |
| --- | --- |
| 2. Adult/ or Middle Aged/ or Aged/ or hospitalized.mp. or Hospitalization/ |  |
| 3. Adult/ or Risk/ or Middle Aged/ or Risk Factors/ or high risk.mp. or Aged/ |  |
| 4. Middle Aged/ or Risk Factors/ or Adult/ or at risk.mp. or Aged/ |  |
| 5. 1 or 2 or 3 or 4 |  |
| 6. patients.mp. or Patients/ |  |
| 7. inpatients.mp. or Inpatients/ |  |
| 8. 6 or 7 |  |
| 9. 5 and 8 |  |
| 10. clinical practice guidelines.mp. or Practice Guideline/ |  |
| 11. evidence based practice.mp. or Evidence-Based Practice/ |  |
| 12. bundles.mp. or Patient Care Bundles/ |  |
| 13. pathways.mp. |  |
| 14. Guideline/ or guidelines.mp. |  |
| 15. 10 or 11 or 12 or 13 or 14 |  |
| 16. Thrombosis/ or venous thromboembolism.mp. or Thrombophlebitis/ or Venous Thromboembolism/ or Thromboembolism/ or Pulmonary Embolism/ |  |
| 17. Thromboembolism/ or Venous Thrombosis/ or Venous Thromboembolism/ or Risk Factors/ or VTE.mp. or Pulmonary Embolism/ |  |
| 18. Pulmonary Embolism/ or Venous Thromboembolism/ or Thromboembolism/ or VTE prophylaxis.mp. or Venous Thrombosis/ |  |
| 19. 16 or 17 or 18 |  |
| 20. 15 and 19 |  |
| 21. Venous Thrombosis/ or Antibiotic Prophylaxis/ or prophylaxis.mp. |  |
| 22. prophylactic.mp. |  |
| 23. prevent.mp. |  |
| 24. reduction.mp. |  |
| 25. decrease.mp. |  |
| 26. diminish.mp. |  |
| 27. 21 or 22 or 23 or 24 or 25 or 26 |  |
| 28. adherence.mp. or Guideline Adherence/ |  |
| 29. Compliance/ or compliance.mp. |  |
| 30. 28 or 29 |  |
| 31. 27 and 30 |  |
| 32. 9 and 20 and 31 |  |
| 33. from 32 keep 1-978 |  |

**Scopus Database**

| TITLE-ABS-KEY ( medical )  OR  TITLE-ABS-KEY ( hospitalized )  OR  TITLE-ABS-KEY ( at  AND risk )  AND  TITLE-ABS-KEY ( patients )  OR  TITLE-ABS-KEY ( inpatients )  AND  TITLE-ABS-KEY ( clinical  AND practice  AND guidelines )  OR  TITLE-ABS-KEY ( evidence  AND based  AND practice )  OR  TITLE-ABS-KEY ( bundles )  OR  TITLE-ABS-KEY ( pathways )  OR  TITLE-ABS-KEY ( protocols )  OR  TITLE-ABS-KEY ( guidelines )  AND  TITLE-ABS-KEY ( venous  AND thromboembolism )  OR  TITLE-ABS-KEY ( vte )  OR  TITLE-ABS-KEY ( vte  AND prophylaxis )  AND  TITLE-ABS-KEY ( prophylaxis )  OR  TITLE-ABS-KEY ( prevent )  OR  TITLE-ABS-KEY ( reduction )  OR  TITLE-ABS-KEY ( decrease )  OR  TITLE-ABS-KEY ( diminish )  OR  TITLE-ABS-KEY ( prophylactic )  AND  TITLE-ABS-KEY ( adherence )  OR  TITLE-ABS-KEY ( compliance ) ) |
| --- |

Cochrane

earch Name: DHA VTE guidelines

Date Run: 12/01/2019 15:32:29

Comment:

ID Search Hits

#1 "Medical":ti,ab,kw (Word variations have been searched)

#2 "Hospitalized":ti,ab,kw (Word variations have been searched)

#3 "High-Risk":ti,ab,kw (Word variations have been searched)

#4 "at risk":ti,ab,kw (Word variations have been searched)

#5 patient*:ti,ab,kw (Word variations have been searched)

#6 inpatient*:ti,ab,kw (Word variations have been searched)

#7 Clinical practice guideline*:ti,ab,kw (Word variations have been searched)

#8 "evidence based practice":ti,ab,kw (Word variations have been searched)

#9 bundle*:ti,ab,kw (Word variations have been searched)

#10 pathway*:ti,ab,kw (Word variations have been searched)

#11 protocol*:ti,ab,kw (Word variations have been searched)

#12 guideline*:ti,ab,kw (Word variations have been searched)

#13 venous thromboembolism:ti,ab,kw (Word variations have been searched)

#14 VTE:ti,ab,kw (Word variations have been searched)

#15 VTE prophylaxis:ti,ab,kw (Word variations have been searched)

#16 prophylaxis:ti,ab,kw (Word variations have been searched)

#17 prevent*:ti,ab,kw (Word variations have been searched) 174948

#18 reduction:ti,ab,kw (Word variations have been searched)

#19 decrease:ti,ab,kw (Word variations have been searched)

#20 diminish:ti,ab,kw (Word variations have been searched)

#21 prophylactic:ti,ab,kw (Word variations have been searched)

#22 adherence:ti,ab,kw (Word variations have been searched)

#23 compliance:ti,ab,kw (Word variations have been searched)

#24 #1 or #2 or #3 or #4 or #5

#25 #5 or #6

#26 #24 and #25

#27 #7 or #8 or #9 or #10 or #11 or #12

#28 #13 or #14 or #15

#29 #27 and #28

#30 #16 or #17 or #18 or #19 or #20 or #21

#31 #22 or #23

#32 #30 and #31

#33 #26 and #29 and #31

Pubmed

((((((((medical*) OR hospital*) OR high-risk*) OR at risk*)) AND ((patient*) OR in patient*))) AND ((((((((clinical practice guideline*) OR evidence based practice*) OR bundle*) OR pathway*) OR protocol*) OR guideline*)) AND (((venous thromboembolism*) OR VTE) OR VTE prophylaxis*))) AND (((((((((prophylaxis*) OR prevent*) OR reduction*) OR decrease*) OR diminish*) OR prophylactic))) AND ((adherence*) OR compliance*))

CINAHL

| **#** | **Query** |
| --- | --- |
| S1 | medical* OR hospital* OR high-risk* OR at risk* |
| S2 | patient* OR in-patient* |
| S3 | clinical practice guideline* OR evidence based practice* OR bundle* OR pathway* OR protocol* OR guideline* |
| S4 | venous thromboembolism* OR VTE OR vte prophylaxis* |
| S5 | S3 AND S4 |
| S6 | prophylaxis* OR prevent* OR reduction* OR decrease* OR diminish* OR prophylactic* |
| S7 | adherence* OR compliance* |
| S8 | S6 AND S7 |
| S9 | S1 AND S2 |
| S10 | S5 AND S8 AND S9 |
| S11 | S5 AND S8 AND S9 |
