# Supplementary material for: Prevention of health care associated venous thromboembolism through implementing VTE prevention clinical practice guidelines in hospitalized medical patients: a systematic review and meta-analysis
Source: Implement Sci. 2020 Jun 24;15:49. doi: 10.1186/s13012-020-01008-9 (PMC7315522; doi:10.1186/s13012-020-01008-9)
Supplement: Supplementary file 4 — Additional file 4. Characteristics of included studies. [file 13012_2020_1008_MOESM4_ESM.docx]

**Characteristics of included studies:**

| **First author (year) Country** | **Study Design** | **Setting/participants** | **Measurement**  **(Guideline Followed, risk assessment tool, intervention)** | **Primary Outcome Measure(s)** | **Other related outcomes** | **Overall conclusion** |
| --- | --- | --- | --- | --- | --- | --- |
| 1. *Duff 2011*   metropolitan Sydney Australia | - Pre and post - Study Period: Sep 2008-Aug 2009 | - 250-bed acute-care private hospital - Inclusion Criteria: Medical and Surgical patients - Included:149 Patients, 50 medical patients - Results reported for medical patients separately - Implementation period 1 year | - The Australian and New Zealand Best Practice Guidelines 4th ed (2007) - An assessment of barriers to VTE guideline was done based on brainstorming and literature review   Selected strategies:   - Audit and feedback - Documentation and decision support aids - Alerts and reminders - Provider education - Hospital VTE prevention Policy/procedure | - % of medical patients receiving appropriate VTE prophylaxis :   19% (4/21) before  44.8% (13/29) after   - % of medical patients with VTE risk assessment: 0% (0/21) Before   3.4 % (1/29) After | - % of All high-risk medical patients receiving appropriate VTE prophylaxis:   15 % (3/20) before 31.6% (6/19) after | - Multifaceted improvement strategy can result in significantly improved rates of VTE prophylaxis and risk assessment - A specifically targeted intervention may be required to improve medical patient prophylaxis |
| 1. *Cardoso, 2016*   *São Paulo, Brazil* | - Pre and post - Study Period: 2010- 2014 | - 450-bed hospital - Inclusion Criteria: clinical or surgical adult patients - Included : 56,834 admissions , clinical, 48.5% (29,172 Clinical admissions), 51.5% (Surgical) - Mar – Dec 2010 (10M) Pre & implementation period, Jan 2011-Mar 2014 (post) | - 8th American college of chest physician (ACCP) Clinical Practice Guidelines   A multiple-strategy approach   - Scientific meetings - Practical training on computerized tools - Continuous training program - Continuous audit and feedback - An institutional protocol for VTE | - % of medical patients with VTE risk assessment within 24 hours of admission   Results 2010- 2014:  90.14% (4,624/5,042)  92.70%(6,593/6,976)  94.5%(5,361/5,611)  93.73% (4,437/4,637) 94.21%(6,840/6,906) | - % of medical patients- days with recommended prophylaxis prescribed   Results 2010- 2014:  62%, 72%, 75%,78%, 78%   - % of discharged patients with a primary or secondary diagnosis of VTE   Results 2010- 2014:  2.03,2.14,1.84,1.62,1.77,  1.69 | - a multiple-strategy approach achieved high rates of   risk assessment within 24 hours of admission, improved the adherence to prophylaxis in high-risk patients |
| 1. Kalili, 2010   Tehran, Iran | - Pre and post study - Study period: Nov 2008 - Aug 2009 | - Teaching hospital - Inclusion Criteria: patients admitted to medical ward - Included: 186 (before), 154 (After), 72 (38.7%) were classified as moderate and high risk patients - 4 months Pre-intervention, 4 months Post-intervention | - Consensual guideline prepared internally - Risk points for thrombosis risk assessment were determined based on institutional agreement - A meeting with the relevant health care professionals and illustrating the results of internal guideline implementation | - % of medical patients who had indication for DVT prophylaxis and anticoagulants were administered: 20.4% (38/186) before 31.2 % (48/154) after | - % of medical patients who had indication for DVT prophylaxis but anticoagulants were not administered: 18.3% (34/186) Before 8.5% (13/154) After - % of patients who had no indication for DVT prophylaxis and anticoagulants were not administered: 49.5 % (92/186) Before 57.2 % (88 /154) After - % of patients who had no indication for DVT prophylaxis but anticoagulants were administered: 11.8% (22/186) Before 2.7 % (5 /154) After | - The implementation of prepared guideline helped to a great extent in improvement of administrating DVT prophylaxis |
| 1. Scaglione 2005   Turin Italy | - Pre and post study - Study period: Jan 2000 - Jun 2002 | - 1500-bed teaching hospital - Inclusion Criteria: medical and surgical patients - Included: 237(before), 71 General medical, 28 medical specialties. 241(After), 98 General medical, 22 medical specialties - Baseline Jan-Jun 2000 (6 months) before intervention, with 18 months implementation period; adoption phase Jan- Jun 2002 same types of patients | - Locally adapted guidelines based on the American College of Chest Physicians (ACCP) guidelines 2001 recommendations - Guidelines was reviewed by three external experts and   heads of department.  Strategies followed:   - Presentation to all hospital physicians - Pocket version of the guideline - multidisciplinary working group to identify possible barriers - Reminders | - % of medical patients receiving appropriate VTE prophylaxis without contraindications to heparins   Low-medium risk:  13.7 % (10/ 73) before,  20% (13/ 65) after,  High risk medical  patients: 25% (5/20)  before, 66.7% (14/21)  after  % of patients who received prophylaxis with contraindication to heparins (12/40) before, (24/57) after | - Number of patients who developed VTE (medical & surgical patients) : (5/237) before, (5/241) after - VTE trend (medical patients):   Number of VTE events/ total number of discharges : 618/ 42722 before 730/ 53595 after | - Implementing a locally adapted evidence-based guidelines may be highly effective in improving appropriateness   of prophylaxis   - Overuse of heparin should be considered as a potential side effect of the guidelines, requiring further intervention |
| 1. *Shedd 2008*   Northeast Georgia | - Pre and post study | - 315-bed community hospital - Inclusion Criteria: medical and surgical patients - Included: 298 patients, 116 medical (before) 74 medical (after) - 5 weeks pre, 5 weeks post only medical patients were included | - VTE Prophylaxis regimens adapted from Caprini’s VTE Risk Assessment tool - The Thrombosis Risk Assessment Tool “Caprini” was used with the permission of Dr. Caprini   The VTE risk evaluation document was placed in the patient’s chart | - % of medical patients receiving appropriate VTE prophylaxis: 43% (50/116) before 76% (56/74) after | - % of medical patients receiving Suboptimal VTE prophylaxis: 9% (10/116) before   5% (4/74) after   - % of medical patients who had no orders for any VTE prophylaxis:   48% (56/116) before 19% (14/74) after | - Thrombosis Risk Assessment Tool was a valuable intervention for improving VTE prophylaxis - An increase in appropriate VTE prophylaxis was observed after a system-level intervention |
| 1. Pai 2013   Canada | - RCT - Cluster - Unit of randomization: groups of 6 hospitals - Unit of analysis: patient - Study period: Jan- Apr 2009 | - 6 hospitals - Inclusion Criteria: all patients ≥18 years of age admitted to the service of general internal medicine - Included: 1154 (intervention) 1457 (control) | - 8th American college of chest physician Clinical Practice Guidelines on Antithrombotic Therapy   Multi component knowledge translation intervention   - Clinician education - Paper based VTE risk assessment algorithm - Printed physicians orders - Audit and feedback session | - % of medical patients appropriately managed for prophylaxis within 24 hours of admission:   66.6% (970/1,457)  Control  64.5% (744/1,154)  intervention | - % of medical patients subject to errors of commission/ administered prophylaxis when unnecessary: 10 % (145/ 1457) Control 9% (105/ 1,154) intervention - % of patients subject to errors of omission/ not administered prophylaxis when necessary: 23% (342/ 1457) Control 26% (305/ 1,154) intervention | - Hospitals allocated to the multi component strategy did not have a higher rate of medical inpatients appropriately managed for prophylaxis |
| 1. Rashid 2005   UK | A prospective survey   - Study period: from Jan 2003 (2 years) | Two NHS teaching  hospitals   - Inclusion Criteria: 1062 medical patients - Included: medical patients - Implementation period(6 Months) | - Guidelines compared with the recommendations of   the THRIFT and ACCP (American College of Chest  Physicians)   - Risk stratified according to the   THRIFT(thromboembolic risk factor) consensus group   - Simple grand-round presentation of the data and recommended guidelines to clinicians | - % of medical patients at moderate or high risk receiving correct VTE prophylaxis based on THRIFT: 4.4% (35/791) before 30.7% (47/153) after - % of medical patients receiving correct VTE prophylaxis based on ACCP : 21.7% (172/791) before 30.7% (47/153) after | - % of medical patients receiving incorrect VTE prophylaxis based on THRIFT: 95.6% (756/791) before 69.3% (106/153) after - % of medical patients receiving incorrect VTE prophylaxis based on ACCP : 78.3 % (619/791) before 69.3 % (106/153) after - % of high risk -medical patients receiving appropriate/correct VTE prophylaxis based on ACCP : 21.7% (172/791) before 30.7% (47/153) after |  |
| 1. Vaughan2011   UK | - Pre and post study | - Inclusion Criteria: all patients in the medical assessment unit - Included:122 (1st round) 101 (2nd round) 163 (3rd round) - Data collection occurred for 14 consecutive days at zero, six and 52 weeks - Implementation period(1 year) | - THRIFT Consensus group (Risk factors for VTE) and the Scottish Intercollegiate   Guidelines Network (SIGN ) 62 (Contraindications and cautions to thromboprophylaxis)   - Risk factors for VTE based on THRIFT Consensus group ( - A local guideline was developed for thromboprophylaxis - addition of a risk assessment to the clerking pro forma - audit - education of juniors | - % of Eligible medical Patients at moderate or high risk who were prescribed prophylactic LMWH : 31 % (10/32) 33 % (10/30) 63% (42) | - % of medical Patients at moderate or high risk who were prescribed TED stockings in where LMWH is contraindicated :   23% (5) 32% (7) 45 %(13)   - % of High-risk medical Patients who were correctly prescribed either TED stockings, LMWH or both : 22 % (8/36) 43% (9/21) 62% (26/42) - % of contraindications to thromboprophylaxis recorded in the notes:   0% (0) 35% (8) 59%  (19) | evidence for the role of audit, education and a risk assessment in improving compliance |
