# Supplementary material for: Prevention of health care associated venous thromboembolism through implementing VTE prevention clinical practice guidelines in hospitalized medical patients: a systematic review and meta-analysis
Source: Implement Sci. 2020 Jun 24;15:49. doi: 10.1186/s13012-020-01008-9 (PMC7315522; doi:10.1186/s13012-020-01008-9)
Supplement: Supplementary file 5 — Additional file 5. Reasons for excluded studies. [file 13012_2020_1008_MOESM5_ESM.docx]

**Characteristics of excluded studies**

|  | **Study** | **Reason for exclusion** |
| --- | --- | --- |
|  | Batman 2013 | Data not analyzed separately for medical patients |
|  | Biffl 2011 | Not study design of interest |
|  | Child 2011 | Poster presentation |
|  | Clark 2011 | Not study design of interest |
|  | DEHEINZELIN 2006 | Not study design of interest |
|  | Faioni 2013 | Poster presentation |
|  | Golian 2014 | abstract only |
|  | Golian 2016 | Not intervention of interest |
|  | Gussoni 2009 | Not study design of interest |
|  | Jenkins 2018 | Not study design of interest |
|  | Jimenez 2017 | Not intervention of interest |
|  | Kalantari 2016 | Not study design of interest |
|  | Khan 2011 | abstract only |
|  | Kim 2015 | Not intervention of interest |
|  | Patil 2016 | abstract only |
|  | Piazza 2009 | Not intervention of interest |
|  | Sad 2014 | abstract only |
|  | Schleyer 2011 | Not study design of interest |
|  | Schleyer 2016 | Not population of interest |
|  | Shah 2012 | Citation Only |
|  | Shah 2010 | Citation Only |
|  | Sharif-Kashani 2012 | Not Population of interest |
|  | Steier 2006 | Not study design of interest |
|  | Thavarajah 2012 | Not population of interest |
|  | Verdu 2009 | Not intervention of interest |
|  | Waltering 2007 | Non- English |
|  | Warcel 2011 | Poster presentation |
|  | Wilson 2011 | Citation Only |
|  | Zeitoun 2009 | Not study design of interest |

Five studies were excluded because the aim of the study was not the implementation of the VTE clinical practice guidelines, thus not an intervention of interest; nine studies were excluded because their study design was not of interest; three studies were not targeting medical patients; Data in one study was not analyzed separately for medical patients; one study was not in English; three studies were poster presentations; three studies were only citations ; and four studies did not provide full data. Several authors were contacted to get the unavailable data, feedback was received from only one study Cardoso that was included in the review and data was utilized in the analysis.
