# Supplementary material for: Prevention of health care associated venous thromboembolism through implementing VTE prevention clinical practice guidelines in hospitalized medical patients: a systematic review and meta-analysis
Source: Implement Sci. 2020 Jun 24;15:49. doi: 10.1186/s13012-020-01008-9 (PMC7315522; doi:10.1186/s13012-020-01008-9)
Supplement: Supplementary file 6 — Additional file 6. MINORS Summary of risk of bias. [file 13012_2020_1008_MOESM6_ESM.docx]

**MINORS a methodological index for non-randomized studies**

MINORS contains 12 methodological points; the first eight apply to both non-comparative and comparative studies, while the remaining four apply only to studies with comparison, including two or more groups. Risk of bias was assessed according to the following criteria: a clearly stated aim; inclusion of consecutive patients; prospective collection of data; endpoints appropriate to the aim of the study; unbiased assessment of the study endpoint; follow-up period appropriate to the aim of the study; loss to follow up less than 5%; and prospective calculation of the study size. Additional criteria in the case of comparative studies; an adequate control group; contemporary groups; baseline equivalence of groups; and adequate statistical analyses.

Each domain was scored 0 if not reported; 1 when reported but inadequate; and 2 when reported and adequate. The global ideal score was 16 for non-comparative studies and 24 for comparative studies. Studies fulfilling all criteria, with full MINORS score, were considered to have low risk bias and high risk of bias was considered in studies with incomplete score.

**Risk of Bias: Individual MINORS score**

***Prevention of health care associated venous thromboembolism through implementing VTE prevention clinical practice guidelines in hospitalized medical patients: A systematic review and meta-analysis***

|  | Duff  2011 | Cardoso  2016 | Kalili 2010 | Scaglione 2005 | Rashid 2005 | Shedd 2008 | Vaughan 2011 |
| --- | --- | --- | --- | --- | --- | --- | --- |
| A clearly stated aim | **2** | **2** | **2** | **2** | **2** | **2** | **2** |
| Inclusion of consecutive patients | **2** | **2** | **2** | **2** | **2** | **2** | **2** |
| Prospective collection of data | **2** | **2** | **2** | **2** | **2** | **2** | **2** |
| Endpoints appropriate to the aim of the study | **2** | **1** | **1** | **1** | **1** | **2** | **1** |
| Unbiased assessment of the study endpoint | **0** | **1** | **1** | **1** | **0** | **2** | **1** |
| Follow-up period appropriate to the aim of the study | **2** | **2** | **2** | **2** | **1** | **2** | **2** |
| Loss to follow up less than 5% | **0** | **0** | **0** | **1** | **1** | **2** | **1** |
| Prospective calculation of the study size | **2** | **2** | **2** | **2** | **1** | **2** | **2** |
| An adequate control group | NA | NA | NA | NA | NA | NA | NA |
| Contemporary groups | NA | NA | NA | NA | NA | NA | NA |
| Baseline equivalence of groups | NA | NA | NA | NA | NA | NA | NA |
| Adequate statistical analyses | NA | NA | NA | NA | NA | NA | NA |
| TOTAL | **12/16** | **12/16** | **12/16** | **13/16** | **10 /16** | **16/16** | **13/16** |

Each domain is scored 0 if not reported; 1 when reported but inadequate; and 2 when reported and adequate
